# Torque Control for a Novel Non-Contact Piezoelectric Motor Modulated by Electromagnetic Force

**DOI:** 10.3390/mi17060718

**Published:** 2026-06-13

**Authors:** Tingting Wang, Moran Xu, Zan Liu

**Affiliations:** College of Mechanical Engineering and Automation, Liaoning University of Technology, Jinzhou 121001, China; xumr@lnut.edu.cn (M.X.); 13870832546@163.com (Z.L.)

**Keywords:** piezoelectric motor, non-contact, torque, PI control, compensation

## Abstract

A novel non-contact piezoelectric motor modulated by electromagnetic force is proposed in this work. The motor consists of a driving system and a transmission system. The transmission system includes a driving torque modulation mechanism and a keeping torque modulation mechanism. The calculation model of the magnetic forces of the motor is deduced, based on which the calculated equations of the magnetic driving torque, the magnetic keeping torque, the total torque, and the torque fluctuation applied to the rotor are presented. The transfer functions of the motor torque and its proportional-integral (PI) control are also given. Compensation control is used to remove the torque fluctuation. Via the derived equations, the effects of the system parameters on the system gain and time constant are investigated. Moreover, the step responses of the motor torque and the effects of the system parameters on them are analyzed, as are the step responses of the closed-loop control system with a PI controller. Furthermore, the torque fluctuation of the rotor is investigated, and its compensation signals are determined. Finally, the compensation control of the torque fluctuation is realized by adding feedback compensation signals.

## 1. Introduction

Piezoelectric motors can be categorized as either contact or non-contact motors according to the contact mode between the stator and rotor [1,2,3,4]. Contact piezoelectric motors transmit motion and power via friction between the stator and rotor working surfaces, and due to wear of the contact surfaces, the service life of the motor is reduced. This prompted the proposal and study of non-contact piezoelectric motors [5,6,7,8].

In 1990, Nakamura proposed a non-contact piezoelectric motor that used liquid to transfer motion, and the maximum measured speed was 50 rpm [9]. In 1996, Nakamura et al. further produced a non-contact rotary piezoelectric motor using electrorheological fluid technology as the transmission medium [10]. In 2011, Yamayoshi et al. proposed a non-contact piezoelectric motor using sound flow as the transmission medium; when using a single-disk rotor, the maximum measured speed was about 2300 rpm, and when using a double-disk rotor, the maximum speed was about 2800 rpm [11,12]. In 2012, Stepanenko et al. investigated a non-contact piezoelectric motor driven by standing waves, which was composed of an annular stator with bending vibration and an asymmetric rotor with blades, and the motor speed was found to be 395 rpm [13]. In the same year, Wang et al. investigated a non-contact piezoelectric actuator characterized by the axial/radial coupling of a spherical rotor, which can provide suspension force and driving force for the spherical rotor along the axial and radial directions, respectively [14]. In 2014, Qiu et al. proposed a bidirectional non-contact rotary piezoelectric motor that combines a piezoelectric torsional vibrator with an electrorheological liquid. The ideal performance of the motor was found to be generated at the electric field intensity of 2 kV/mm and the duty cycle of 30%, at which the maximum speed of the motor was 6.98 rad/s and the torque was 1.04 N·mm [15]. In 2019, Hirano et al. applied an acoustic viscous force to a non-contact piezoelectric motor, which could realize continuous rotation by switching the driving stator [16]. In the same year, Zhang et al. proposed a non-contact rotary piezoelectric motor driven by surface acoustic waves, with a higher rotational speed and lower threshold of driving voltage [17]. In 2020, Xing et al. proposed the use of electromagnetic force as the transmission medium of a non-contact piezoelectric motor. It was found that when the electromagnetic force was parallel to the radial direction of the motor, the motor speed was about 0.038 rad/s, and the output torque was about 6 N·mm; when the electromagnetic force was parallel to the axial direction of the motor, the motor speed was about 0.1046 rad/min, and the output torque was about 0.405 N·mm [18,19]. In 2021, Shi et al. proposed an adaptive non-contact piezoelectric motor that does not require a phase control system; the rotor motion is realized via the high-frequency tilted vibration of four drive units, and the torque is generated by acoustic radiation caused by the asymmetric modal vibration [20,21,22]. In 2025, Li et al. proposed a non-contact piezoelectric motor that uses compressed air as a transmission medium to generate high pressure and drive the rotor to rotate [23].

In these non-contact piezoelectric motors using electromagnetic force as the transmission medium (such as Refs. [18,19]), there are no special requirements for the frequency of the excitation signal, so they are easier to use in engineering. However, they adopt a single electromagnetic modulation mechanism and a flexible amplification mechanism. The single mechanism lacks a holding force when the driving voltage is zero, and the flexible mechanism results in significant power loss and low output torque [24].

The output torque is a key factor for the measurement of the operating performance of a motor, and depends on the size, structure, and material of the motor [25,26]. Therefore, a non-contact piezoelectric motor using electromagnetic force and a rigid amplification mechanism has been proposed [27]. However, it still uses a single electromagnetic modulation mechanism. This motor is driven by discontinuous electromagnetic torque under the pulse voltage, which easily causes the rotor to rotate in reverse, thereby limiting its load-carrying capacity.

To address the above issues, a novel non-contact piezoelectric motor modulated by electromagnetic force is proposed in this paper. It includes two electromagnetic modulation mechanisms, namely a driving torque modulation mechanism and a keeping torque modulation mechanism. The newly introduced keeping mechanism ensures the rotor remains stationary during pulse intervals, thereby avoiding the reverse rotation under zero voltage. The fluctuation of the output torque occurs in the motor. Nevertheless, the good dynamic performance of the motor can be realized by a control technique [28,29]. Thus, to eliminate the torque fluctuation, a torque control model is developed. The calculation model of the magnetic forces of the motor is deduced, and the calculated equations of the magnetic driving torque, the magnetic keeping torque, the total torque, and the torque fluctuation of the motor are presented. The transfer functions of the motor torque and fluctuation compensation control via a proportional-integral (PI) controller are given. Using these equations, the effects of the system parameters on the system gain and time constant are investigated, and the step responses of the motor torque are analyzed. Moreover, the step responses of the closed-loop control system with a PI controller are studied, as are the torque fluctuation of the motor and its compensation control. The simulation shows that the torque fluctuation is effectively suppressed, and a good dynamic response of the motor is achieved, providing a foundation for subsequent prototype development and experimental validation.

## 2. Motor Structure and Working Principle

**Figure 1:**
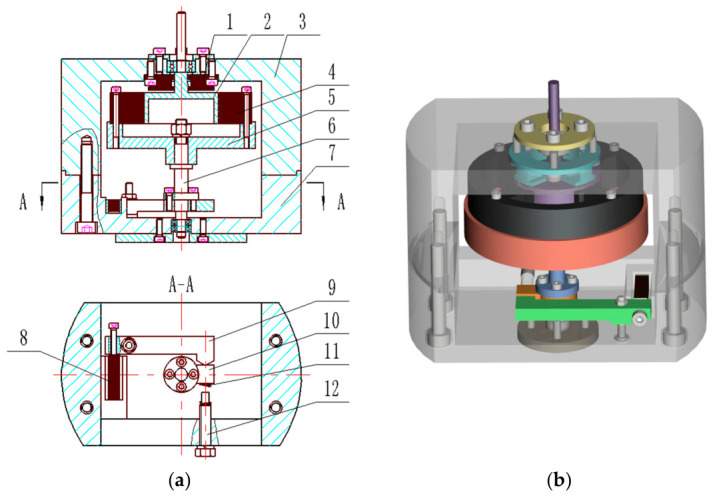
Non-contact piezoelectric motor modulated by electro-magnetic forces: (**a**) schematic diagram; (**b**) 3D model; 1—keeping torque modulation mechanism(cyan); 2—hollow rotor(purple); 3—cover; 4—driving torque modulation mechanism(black); 5—base(orange-red); 6—shaft(blue); 7—lower cover; 8—piezoelectric stack; 9—swinging rod(green); 10—beam(orange); 11—adjustment spring; 12—thread rod.

The non-contact piezoelectric motor modulated by electromagnetic force consists of a driving system and a transmission system; the driving system includes a piezoelectric stack, a swinging rod, a beam, a threaded rod, and an adjustable spring, while the transmission system includes a shaft, a chassis, two electromagnetic modulation mechanisms, and a hollow rotor. The two electromagnetic modulation mechanisms are the driving torque modulation mechanism and the keeping torque modulation mechanism (see Figure 1).

**Figure 2:**
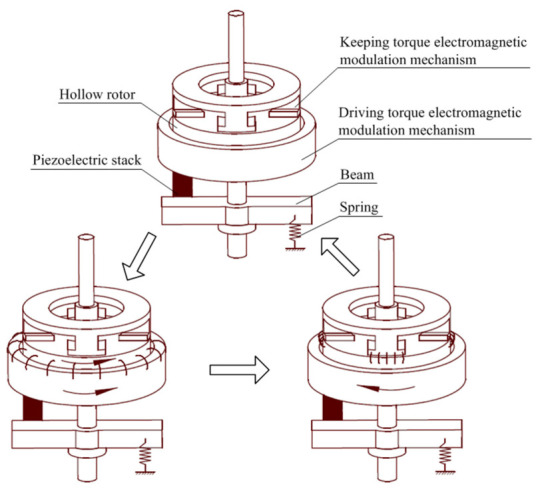
Working process of the non-contact piezoelectric motor.

The working process of the non-contact piezoelectric motor modulated by electromagnetic force is presented in Figure 2.

As the piezoelectric stack is energized, it pushes the swinging rod (9) to move (the displacement of the piezoelectric stack is enlarged), after which the swinging rod pushes the beam (10) to rotate. Simultaneously, the torque is transmitted to the driving torque modulation mechanism (4) through the shaft (6). At this time, the driving torque modulation mechanism generates a magnetic force to attract the hollow rotor (2), thereby driving the hollow rotor to rotate to output the torque and angular displacement. When the piezoelectric stack is powered off, the driving torque modulation mechanism is powered off and is separated from the hollow rotor (2). Simultaneously, the keeping torque modulation mechanism (1) is energized to generate an electromagnetic force to attract the hollow rotor, so the hollow rotor remains in the static state. The beam and swinging rod return to the initial position by the restoring force generated by adjusting the spring (11).

This process is carried out in a cycle; the beam experiences an inverse and clockwise swing, and the hollow rotor undergoes an intermittent counterclockwise rotation, thereby achieving the continuous power output of the rotor.

## 3. Electromagnetic Forces

**Figure 3:**
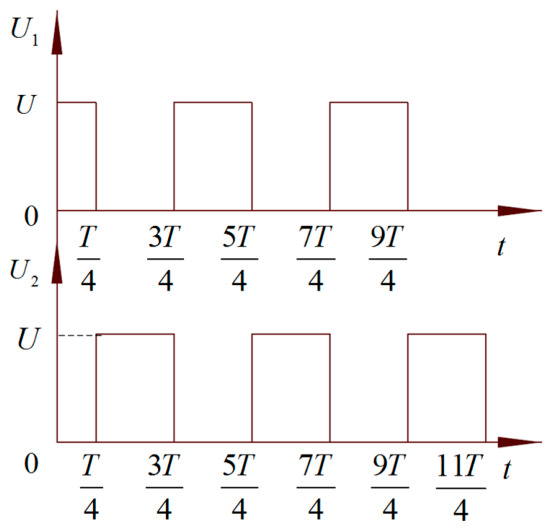
Pulse voltage signals for electromagnetic modulation mechanisms.

The non-contact piezoelectric motor modulated by electromagnetic force comprises a driving torque modulation mechanism and a keeping torque modulation mechanism. The motor undergoes step rotation under a pulsed voltage via the driving torque modulation mechanism, and remains in the static state under a pulsed voltage via the keeping torque modulation mechanism. The pulse voltage signals for the driving torque modulation mechanism (*U*_1_) and the keeping torque modulation mechanism (*U*_2_) are exhibited in Figure 3.

The non-contact piezoelectric motor modulated by electromagnetic force uses a rigid structure, and its operating frequency is far below the mechanical resonance frequency. Consequently, the air gaps between the stators and the rotor can be considered essentially constant during operation, making inductance variations negligible. The rotor is fabricated from a magnetically permeable material with low electrical conductivity, and the motor operates at a relatively low rotational speed. Under these conditions, the inertial forces are much smaller than the electromagnetic driving torque, and the electrodynamic effects induced by the rotor’s motion are extremely weak. Therefore, the electromagnetic forces can be calculated using a static magnetic field model, and the dynamic coupling of the electromechanical system can be neglected.

**Figure 4:**
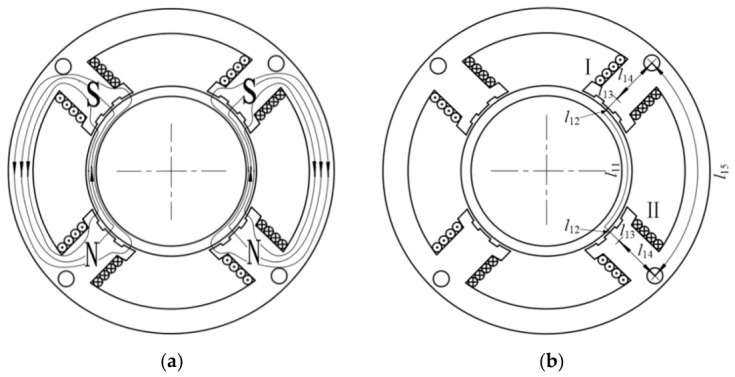
Schematic diagram of driving torque modulation mechanism: (**a**) magnetic induction line distribution; (**b**) magnetic circuits.

**Figure 5:**
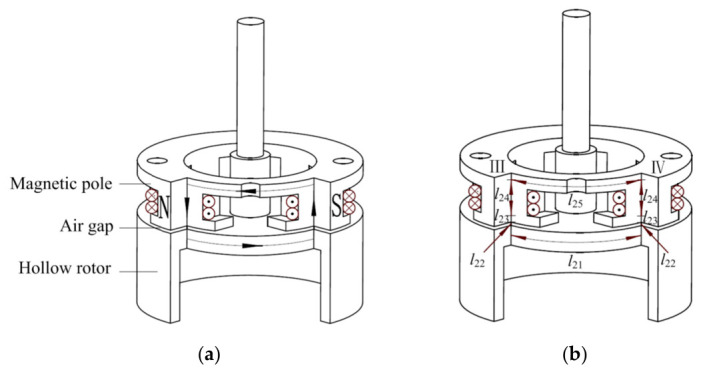
Schematic diagram of the keeping torque modulation mechanism: (**a**) magnetic induction line distribution; (**b**) magnetic circuits.

The electromagnetic modulation mechanism is mainly composed of silicon steel sheets and coil windings; the distributions of the magnetic induction lines in the winding are presented in Figure 4a and Figure 5a, respectively. Ignoring magnetic leakage, one pair of coil windings was selected and divided into five situations for magnetic circuit analysis according to different materials and cross-sectional areas, as shown in Figure 4b and Figure 5b, respectively.

According to Ohm’s law for magnetic circuits, the reluctance of section *l*_11_ on the hollow rotor of the driving torque modulation mechanism can be given by

(1) $$R_{11}=\frac{l_{11}}{\mu_{1}\mu_{0}S_{11}},$$

where *μ*_0_ is the vacuum permeability, *μ*_1_ is the relative permeability of the hollow rotor, *S*_11_ is the cross-sectional area of section *l*_11_ on the magnetic circuit, *S*_11_ = *n_g_t_g_b*_11_, *n_g_* is the number of silicon steel sheets, *t_g_* is the thickness of a single silicon steel sheet, *b*_11_ is the width of section *l*_11_ on the magnetic circuit, *l*_11_ is the average magnetic circuit length of the hollow rotor for the driving torque modulation mechanism, *l*_11_ = π*l*_11_/2, and *r*_2_ is the radius of the hollow rotor.

In the same manner, the reluctances of sections *l*_12_–*l*_15_ on the driving torque modulation mechanism can be given by

$$R_{12}=\frac{l_{12}}{\mu_{2}\mu_{0}S_{12}},\;R_{13}=\frac{l_{13}}{\mu_{r}\mu_{0}S_{13}},\;R_{14}=\frac{l_{14}}{\mu_{r}\mu_{0}S_{14}}\mathrm{and}\;R_{15}=\frac{l_{15}}{\mu_{r}\mu_{0}S_{15}},$$

where $l_{15}=0.5\pi (r_{2}+l_{12}+l_{13}+l_{14})$, *μ*_2_ is the relative permeability of the air gap, and *μ_r_* is the relative permeability of the silicon steel sheet. Moreover, *S*_12_ = *n_g_t_g_b*_12_, *S*_13_ = *n_g_t_g_b*_13_, *S*_14_ = *n_g_t_g_b*_14_, and *S*_15_ = *n_g_t_g_b*_15_, which respectively define the cross-sectional areas of sections *l*_12_–*l*_15_. Finally, *b*_12_–*b*_15_ are the magnetic circuit widths of sections *l*_12_–*l*_15_, respectively.

The reluctance of section *l*_21_ on the hollow rotor of the keeping torque modulation mechanism can be given by

(2) $$R_{21}=\frac{l_{21}}{\mu_{1}\mu_{0}S_{21}},$$

where *S*_21_ is the cross-sectional area of section *l*_21_ on the magnetic circuit, and *l*_11_ is the average magnetic circuit length of the hollow rotor of the keeping torque modulation mechanism.

In the same manner, the reluctances of sections *l*_22_–*l*_25_ on the keeping torque modulation mechanism can be given by

$$R_{22}=\frac{l_{22}}{\mu_{2}\mu_{0}S_{22}},\;R_{23}=\frac{l_{23}}{\mu_{i}\mu_{0}S_{23}},\;R_{24}=\frac{l_{24}}{\mu_{i}\mu_{0}S_{24}}\mathrm{and}\;R_{25}=\frac{l_{25}}{\mu_{i}\mu_{0}S_{25}},$$

where $l_{25}=l_{21}$, and *μ_i_* is the relative permeability of the core.

**Figure 6:**
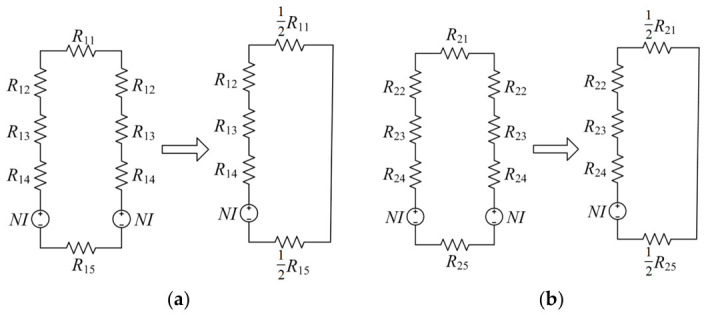
Schematic diagrams of equivalent magnetic circuits: (**a**) driving torque modulation mechanism; (**b**) keeping torque modulation mechanism.

As magnetic poles I and II have the same polarity, they form a series magnetic circuit. For the convenience of calculation, the magnetic circuit is simplified, as shown in Figure 6a. Similarly, the simplified magnetic circuits of magnetic poles III and IV can be obtained, as shown in Figure 6b.

The equivalent total reluctances of the magnetic circuit of the driving torque modulation mechanism and the keeping torque modulation mechanism are, respectively

(3) $$R_{m}=\frac{1}{2}R_{11}+R_{12}+R_{13}+R_{14}+\frac{1}{2}R_{15},$$

(4) $$R_{n}=\frac{1}{2}R_{21}+R_{22}+R_{23}+R_{24}+\frac{1}{2}R_{25}.$$

Because the air gap between the iron core and the hollow rotor in the two electromagnetic modulation mechanisms is very small, and because the corresponding surface can be approximated as parallel to the plane, according to Ohm’s law for magnetic circuits and the simplified Maxwell’s electromagnetic suction equation, the electromagnetic force at a single magnetic pole in the driving torque modulation mechanism is

(5) $$F_{e}=\frac{2N^{2}I^{2}}{\mu_{0}n_{g}t_{g}b_{12}{\left(R_{11}+2R_{12}+2R_{13}+2R_{14}+R_{15}\right)}^{2}}.$$

where *N* is the number of coil turns, and *I* is the current in the coils.

Moreover, the electromagnetic force at a single magnetic pole in the keeping torque modulation mechanism is

(6) $$F_{E}=\frac{2N^{2}I^{2}}{\mu_{0}S_{22}{\left(R_{21}+2R_{22}+2R_{23}+2R_{24}+R_{25}\right)}^{2}}.$$

The electromagnetic force is proportional to the square of the current *I* and inversely proportional to the square of the total reluctance. Decreasing the air-gap length significantly increases the electromagnetic force.

**Figure 7:**
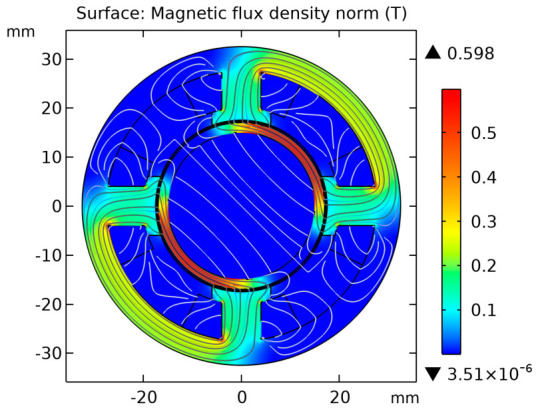
Magnetic flux density nephogram.

To verify the theoretical magnetic field distribution of the driving torque modulation mechanism described above and to clarify the preconditions for torque generation, a finite element analysis was performed using COMSOL Multiphysics 6.2. Figure 7 shows the magnetic flux density distribution of the driving torque modulation mechanism and the hollow rotor at a coil current of 0.2 A. The simulated magnetic field lines form closed loops through the stator, air gaps, and hollow rotor. This flux path is highly consistent with the theoretical magnetic field distribution. Furthermore, the maximum magnetic flux density is approximately 0.598 T, which mainly occurs in the flux concentration region of the hollow rotor. This value is far below the saturation flux density of the stator and rotor materials, ensuring reliable operation of the electromagnetic structure in the linear region without magnetic saturation. This magnetic field generates the normal electromagnetic attraction force required to clamp the rotor. When the piezoelectric stack drives the stator to swing, the hollow rotor, along with the stator, rotates to output the torque and angular displacement.

## 4. Electromagnetic Torques

**Figure 8:**
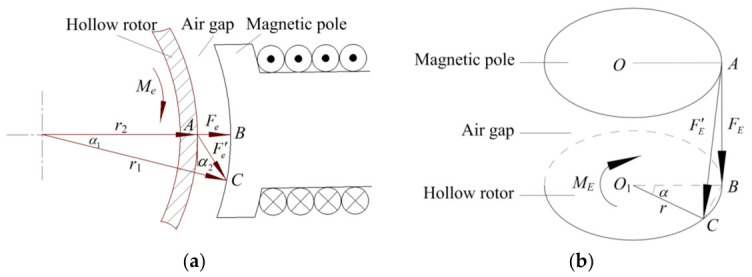
Calculation of electromagnetic torques: (**a**) driving torque; (**b**) keeping torque.

When the input voltage drives the shaft and the electromagnetic modulation mechanism to rotate, due to the inertia of the hollow rotor, its movement lags behind that of the electromagnetic modulation mechanism. Thus, the normal electromagnetic force between the pole and the hollow rotor deviates, which forms a slip angle *α*_1_ (see Figure 8a). In Figure 8a, *F_e_* denotes the electromagnetic force when *α*_1_ = 0, and $F_{e}^{\prime }$ denotes the electromagnetic force when *α*_1_ ≠ 0. Moreover, $r_{1}=r_{2}+l_{12}$, where *r*_1_ is the radius of the hollow rotor and $l_{12}^{\prime }$ is the length of AC in Figure 8a.

From Figure 8a, the following can be known

(7) $$l_{12}^{\prime }=\sqrt{r_{1}^{2}+r_{2}^{2}-2r_{1}r_{2}\cos\alpha_{1}}.$$

Thus, the reluctance of the air gap is

(8) $$R_{12}^{\prime }=\frac{l_{12}^{\prime }}{\mu_{2}\mu_{0}S_{12}}.$$

The total reluctance is

(9) $$R_{m}^{\prime }=\frac{1}{2}R_{11}+R_{12}^{\prime }+R_{13}+R_{14}+\frac{1}{2}R_{15}.$$

By substituting Equation (9) into Equation (5), the magnetic force at *α*_1_ ≠ 0 can be given by

(10) $$F_{e}^{\prime }=\frac{N^{2}I^{2}}{2\mu_{0}n_{g}t_{g}b_{12}{R_{m}^{\prime }}^{2}}.$$

Therefore, the magnetic torque can be given by

(11) $$M_{e}=F_{e}^{\prime }r_{2}\cos\alpha_{2}.$$

By substituting $\cos\alpha_{2}=\frac{r_{1}\sin\alpha_{1}}{l_{12}^{\prime }}$ into Equation (11), the magnetic driving torque for a single pole can be obtained as follows

(12) $$M_{e}=\frac{N^{2}I^{2}r_{1}r_{2}\sin\alpha_{1}}{2\mu_{0}n_{g}t_{g}b_{12}{R_{m}^{\prime }}^{2}{\left(r_{1}^{2}+r_{2}^{2}-2r_{1}r_{2}\cos\alpha_{1}\right)}^{\frac{1}{2}}}.$$

The electromagnetic keeping torque is shown in Figure 8b. In the figure, *α* is the slip angle between the hollow rotor and pole of the keeping modulation mechanism, *F_E_* is the electromagnetic force when *α* = 0, $F_{E}^{\prime }$ is the electromagnetic force when *α* ≠ 0, and *r* is the radius of the hollow rotor of the keeping modulation mechanism. From Figure 8b, the following can be determined

(13) $$l_{22}^{\prime }=\sqrt{l_{22}^{2}+4r^{2}{\left(\sin\frac{\alpha }{2}\right)}^{2}}.$$

Thus, the reluctance of the air gap is

(14) $$R_{22}^{\prime }=\frac{l_{22}^{\prime }}{\mu_{2}\mu_{0}S_{22}}$$

Moreover, the total reluctance is

(15) $$R_{n}^{\prime }=\frac{1}{2}R_{21}+R_{22}^{\prime }+R_{23}+R_{24}+\frac{1}{2}R_{25}.$$

By substituting Equation (15) into Equation (6), the magnetic force at *α* ≠ 0 can be given by

(16) $$F_{E}^{\prime }=\frac{N^{2}I^{2}}{2\mu_{0}S_{22}{R_{n}^{\prime }}^{2}}.$$

Therefore, the magnetic keeping torque for a single pole can be obtained as follows

(17) $$M_{E}=F_{E}^{\prime }r\cos\beta =\frac{2N^{2}I^{2}r^{2}\sin\left(\frac{\alpha }{2}\right)}{2\mu_{0}S_{22}{R_{n}^{\prime }}^{2}{\left(l_{22}^{2}+4r^{2}{\left(\sin\frac{\alpha }{2}\right)}^{2}\right)}^{\frac{1}{2}}},$$

where $\cos\beta =2r\sin\left(\frac{\alpha }{2}\right)/l_{22}^{\prime }$, and *β* is the angle between AC and CB in Figure 8b.

For the driving and keeping torque modulation mechanisms, the input voltage signals are respectively

(18) $$U_{1}\left(t\right)=\frac{U}{2}+\frac{2U}{\pi }\sum_{n=1}^{\infty }\left[\frac{{\left(-1\right)}^{n-1}}{2n-1}\cos\left(2n-1\right)\omega t\right],$$

(19) $$U_{2}\left(t\right)=\frac{U}{2}+\frac{2U}{\pi }\sum_{n=1}^{\infty }\left[\frac{{\left(-1\right)}^{n}}{2n-1}\cos\left(2n-1\right)\omega t\right],$$

where *U*_1_(*t*) and *U*_2_(*t*) are the input voltage signals for the driving and keeping torque modulation mechanisms, respectively, *U* is the peak voltage, *ω* is the frequency of the voltage signals, and the phase difference between the two signals is *T*/2.

The electromagnetic modulation circuit consists of winding resistance and inductance, so it can be equivalent to a resistor–inductor (RL) circuit. According to the voltage relationship in the series circuit, it is known that

(20) $$U_{1}\left(t\right)=L\frac{di_{e}}{dt}+Ri_{e},$$

where *i_e_* is the current in the driving torque modulation mechanism, *L* is the circuit inductance, and *R* is the circuit resistance. Solving this differential equation with the pulsed voltage *U*_1_(*t*) given in Equation (18) and the initial condition *i_e_*(0) = 0, the dynamic current in the driving torque modulation mechanism is (The detailed derivation is provided in Appendix A)

(21) $$\begin{array}{c} i_{e}\left(t\right)=\frac{U}{2R}\left(1-e^{-\frac{R}{L}t}\right)+\sum_{n=1}^{\infty }\frac{{\left(-1\right)}^{n-1}2U}{\left(2n-1\right)\pi \left(R^{2}+{\left(2n-1\right)}^{2}\omega^{2}L^{2}\right)}[R\cos\left(2n-1\right)\omega t+\\ \;\;\;\;\;\left(2n-1\right)\omega L\sin\left(2n-1\right)\omega t-{Re}^{-\frac{R}{L}t}]. \end{array}$$

By substituting Equation (21) into Equation (12), the dynamic magnetic driving torque for a single pole in the driving torque modulation mechanism can be obtained as follows

(22) $$M_{e}(t)=\frac{N^{2}i_{e}{\left(t\right)}^{2}r_{1}r_{2}\sin\alpha_{1}}{2\mu_{0}n_{g}t_{g}b_{12}{R_{m}^{\prime }}^{2}{\left(r_{1}^{2}+r_{2}^{2}-2r_{1}r_{2}\cos\alpha_{1}\right)}^{\frac{1}{2}}}.$$

In the same manner, the dynamic current in the keeping torque modulation mechanism can be given by the following equation

(23) $$\begin{array}{c} i_{E}\left(t\right)=\frac{U}{2R}\left(1-e^{-\frac{R}{L}t}\right)+\sum_{n=1}^{\infty }\frac{{\left(-1\right)}^{n}2U}{\left(2n-1\right)\pi \left(R^{2}+{\left(2n-1\right)}^{2}\omega^{2}L^{2}\right)}\left[R\cos\left(2n-1\right)\omega t+\right.\\ \;\;\;\;\;\left(2n-1\right)\omega L\sin\left(2n-1\right)\omega t-Re^{-\frac{R}{L}t}]. \end{array}$$

By substituting Equation (23) into Equation (17), the dynamic magnetic keeping torque for a single pole in the keeping torque modulation mechanism can be obtained as follows

(24) $$M_{E}(t)=\frac{2N^{2}i_{E}{\left(t\right)}^{2}r^{2}\sin\left(\frac{\alpha }{2}\right)}{2\mu_{0}S_{22}{R_{n}^{\prime }}^{2}{\left(l_{22}^{2}+4r^{2}{\left(\sin\frac{\alpha }{2}\right)}^{2}\right)}^{\frac{1}{2}}}.$$

The total electromagnetic torque of the motor system is the sum of the electromagnetic torques of the two electromagnetic modulation mechanisms, as follows

(25) $$M(t)=M_{E}(t)+M_{e}(t).$$

Additionally, the torque fluctuation is

(26) $$\Delta M(t)=-\left[M(t)-M_{d}(t)\right],$$

where $M_{d}(t)$ is the average electromagnetic torque, $M_{d}(t)=\frac{1}{T}\int_{0}^{T}M(t)dt$.

## 5. Transfer Function from Voltage to Electromagnetic Torque

Based on Equations (22) and (24), the electromagnetic torque is proportional to the square of the current. However, when a large current is used, there is an approximately linear relationship between the torque and current due to the saturation of the magnetic circuit. Thus, Equations (25) and (26) can respectively be changed to the following forms

(27) $$M_{e}(t)=\frac{N^{2}i_{e}\left(t\right)r_{1}r_{2}\sin\alpha_{1}}{2\mu_{0}n_{g}t_{g}b_{12}{R_{m}^{\prime }}^{2}{\left(r_{1}^{2}+r_{2}^{2}-2r_{1}r_{2}\cos\alpha_{1}\right)}^{\frac{1}{2}}},$$

(28) $$M_{E}(t)=\frac{2N^{2}i_{E}\left(t\right)r^{2}\sin\left(\frac{\alpha }{2}\right)}{2\mu_{0}S_{22}{R_{n}^{\prime }}^{2}{\left(l_{22}^{2}+4r^{2}{\left(\sin\frac{\alpha }{2}\right)}^{2}\right)}^{\frac{1}{2}}}.$$

The Laplace transforms for Equations (20) and (27) are

(29) $$M_{e}(s)=\frac{N^{2}I_{e}\left(s\right)r_{1}r_{2}\sin\alpha_{1}}{2\mu_{0}n_{g}t_{g}b_{12}{R_{m}^{\prime }}^{2}{\left(r_{1}^{2}+r_{2}^{2}-2r_{1}r_{2}\cos\alpha_{1}\right)}^{\frac{1}{2}}},$$

(30) $$U_{1}\left(s\right)=LsI_{e}(s)+RI_{e}\left(s\right).$$

Thus, the transfer function from the voltage to the driving electromagnetic torque can be given by

(31) $$G(s)=\frac{M_{e}(s)}{U_{1}\left(s\right)}=\frac{N^{2}r_{1}r_{2}\sin\alpha_{1}}{2\mu_{0}n_{g}t_{g}b_{12}{R_{m}^{\prime }}^{2}\left(Ls+R\right){\left(r_{1}^{2}+r_{2}^{2}-2r_{1}r_{2}\cos\alpha_{1}\right)}^{\frac{1}{2}}}=\frac{K}{Ts+1},$$

where $K=\frac{N^{2}r_{1}r_{2}\sin\alpha_{1}}{2\mu_{0}n_{g}t_{g}b_{12}R{R_{m}^{\prime }}^{2}{\left(r_{1}^{2}+r_{2}^{2}-2r_{1}r_{2}\cos\alpha_{1}\right)}^{\frac{1}{2}}}$, and $T=\frac{L}{R}$.

The transfer function is first-order, with gain *K* and time constant *T*. The time constant *T* is determined solely by the RL circuit; a smaller *T* gives faster torque response. Since the pole *s* = −1/*T* lies on the negative real axis, the system is stable and non-oscillatory.

The transfer function from the voltage to the keeping electromagnetic torque can also be obtained in the same manner.

## 6. Results and Discussion

According to the transfer function model of the non-contact piezoelectric motor drive system, the parameters that affect the dynamic response of the system include both mechanical and electrical parameters. To endow the system with a good dynamic response, it is necessary to study the influences of these two types of parameters on the dynamic response.

### 6.1. Effects of the System Parameters on the System Gain

By expanding the parameters contained in the equivalent total reluctance $R_{m}^{\prime }$ of the magnetic circuit, the relationships between the gain and parameters in the transfer function model of the drive system are obtained as follows:

(32) $$K=\frac{N^{2}\mu_{0}n_{g}t_{g}r_{2}\sin\alpha_{1}\left(r_{2}+l_{12}\right)}{2b_{12}R{\left(\frac{l_{11}}{2\mu_{1}b_{11}}+\frac{l_{12}^{\prime }}{\mu_{2}b_{12}}+\frac{l_{13}}{\mu_{r}b_{12}}+\frac{l_{14}}{\mu_{r}b_{14}}+\frac{l_{15}}{2\mu_{r}b_{15}}\right)}^{2}l_{12}^{\prime }},$$

where $l_{11}=\frac{1}{2}\pi r_{2}$, $l_{12}^{\prime }=\sqrt{{\left(r_{2}+l_{12}\right)}^{2}+r_{2}^{2}-2\left(r_{2}+l_{12}\right)r_{2}\cos\alpha_{1}}$, and $l_{15}=\frac{1}{2}\pi (r_{2}+l_{12}+l_{13}+l_{14})$.

According to Equation (32), the system gain has a simple proportional relationship with the number *n_g_* of silicon steel sheets and the thickness *t_g_* of a single-layer silicon steel sheet; moreover, it is inversely proportional to the resistance *R* of the winding coil.

**Figure 9:**
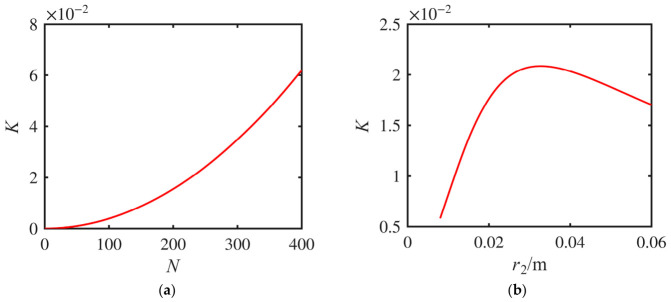

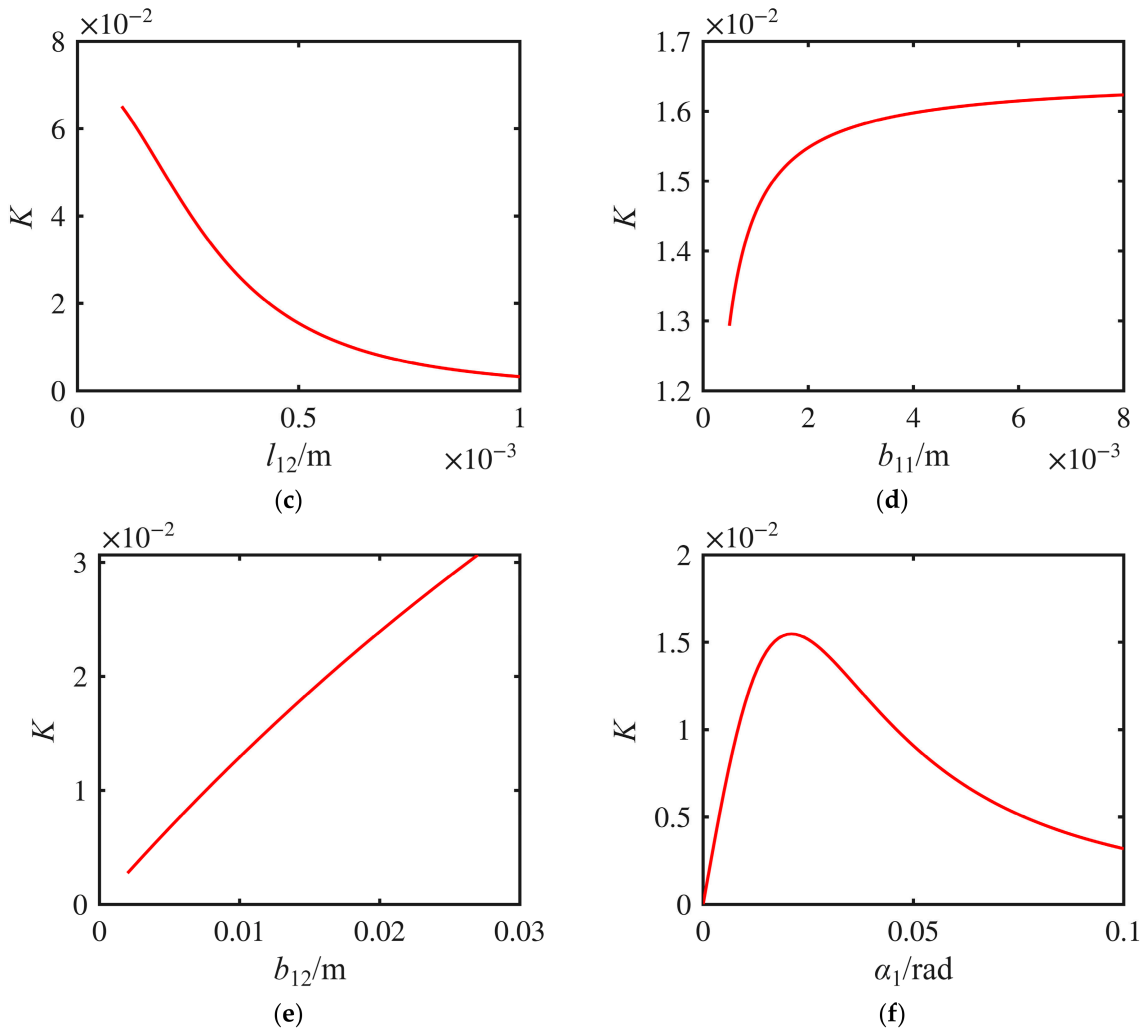
Effects of the system parameters on system gain: (**a**) *N* changes; (**b**) *r*_2_ changes; (**c**) *l*_12_ changes; (**d**) *b*_11_ changes; (**e**) *b*_12_ changes; (**f**) *α*_1_ changes.

The influences of the number of coil turns *N*, the radius *r*_2_ of the hollow rotor, the air gap length *l*_12_, the magnetic circuit widths *b*_11_, *b*_12_, *b*_14_, and *b*_15_, the relative permeability *μ*_1_ of the hollow rotor, the relative permeability *μ_r_* of the silicon steel sheet, the average magnetic circuit lengths *l*_13_ and *l*_14_, and the slip angle *α*_1_ on the system gain were investigated (see Figure 9; due to spatial limitations, only a portion of the results is provided). Figure 9 indicates the following.

(1) The system gain increases with the increase in the number of coil turns *N*, the magnetic circuit width *b*_12_, and the relative permeabilities *μ*_1_ and *μ_r_*. The greater the number of coil turns *N*, the faster the rate at which the gain of the system increases. Moreover, the magnetic circuit width *b*_12_ and the relative permeability of the hollow rotor and silicon steel sheet have an approximately linear relationship with the system gain. Therefore, within a reasonable range, the larger the number of coil turns *N*, the larger the magnetic circuit width *b*_12_, and the larger the relative permeability of the hollow rotor and silicon steel sheet, the larger the system gain.

(2) With the increases in the radius *r*_2_ of the hollow rotor and the slip angle *α*_1_, the system gain first increases, reaches a maximum value, and then decreases. The gain reaches the peak value at *r*_2_ = 32.42 mm and *α*_1_ = 0.021 rad. With the increase in the magnetic circuit width *b*_12_, the system gain gradually increases. However, the magnetic circuit widths *b*_11_, *b*_14_, and *b*_15_ have little effect on the system gain.

(3) With the increases in the air gap length *l_1_*_2_ and the average magnetic circuit lengths *l*_13_ and *l*_14_, the system gain decreases gradually. The smaller the air gap length, the faster the system gain increases. Moreover, the average magnetic circuit lengths *l*_13_ and *l*_14_ are approximately proportional to the system gain, but have little effect on it.

### 6.2. Effects of the System Parameters on the Time Constant

The time constant *T* can affect the dynamic characteristics of the system, so it is an important characteristic parameter of the first-order linear system. When the value of *T* is small, the inertia generated by the system is also small, so the response speed of the system is fast.

The time constant of the non-contact piezoelectric motor can be expressed as follows

(33) $$T=\frac{4\pi \mu_{r}R_{t}N^{2}}{l_{16}R}\left[\pi R_{t}k_{L}-h\left(0.693+C_{L}\right)\right]\times 10^{-7}.$$

According to Equation (33), the time constant *T* has a simple proportional relationship with the relative permeability of the silicon steel sheet. Furthermore, it is inversely proportional to the resistance *R* of the multilayer winding coil, the total length *l*_16_ of the coil, and the thickness *h* of the coil.

**Figure 10:**
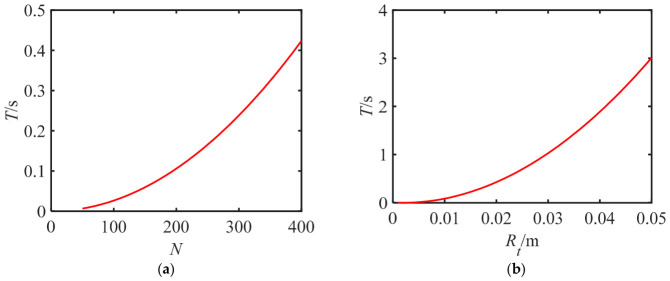
The effects of the system parameters on the time constant: (**a**) *N* changes; (**b**) *R_t_* changes.

The influences of the number of coil turns *N* and the average radius *R*_t_ of the winding on the time constant are shown in Figure 10 (due to space limitations, only a portion of the results is presented). Figure 10 reveals the following.

(1) The time constant increases with the increase in the number of coil turns *N* and the average radius *R_t_*. The time constant increases more quickly with the increases in *N* and *R_t_* for larger values of *N* and *R_t_*.

(2) A smaller time constant can be obtained by decreasing the number of coil turns *N*, the average radius of the winding, and the relative permeability of the silicon steel sheet; a smaller time constant can also be obtained by increasing the resistance, the total length, and the thickness of the winding coil.

### 6.3. Step Responses

**Figure 11:**
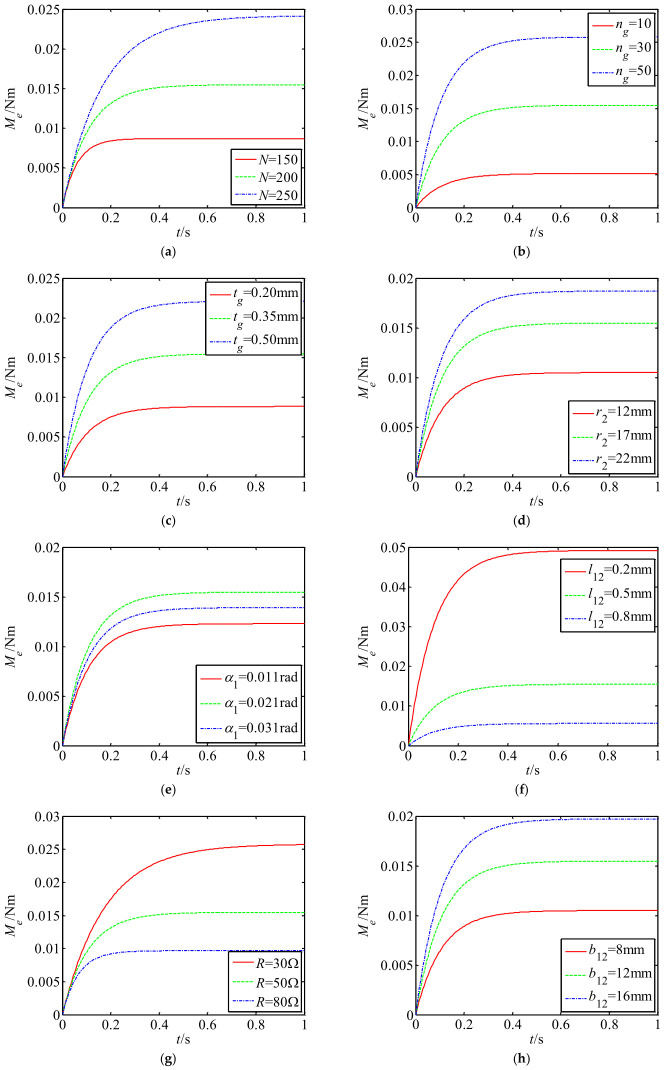

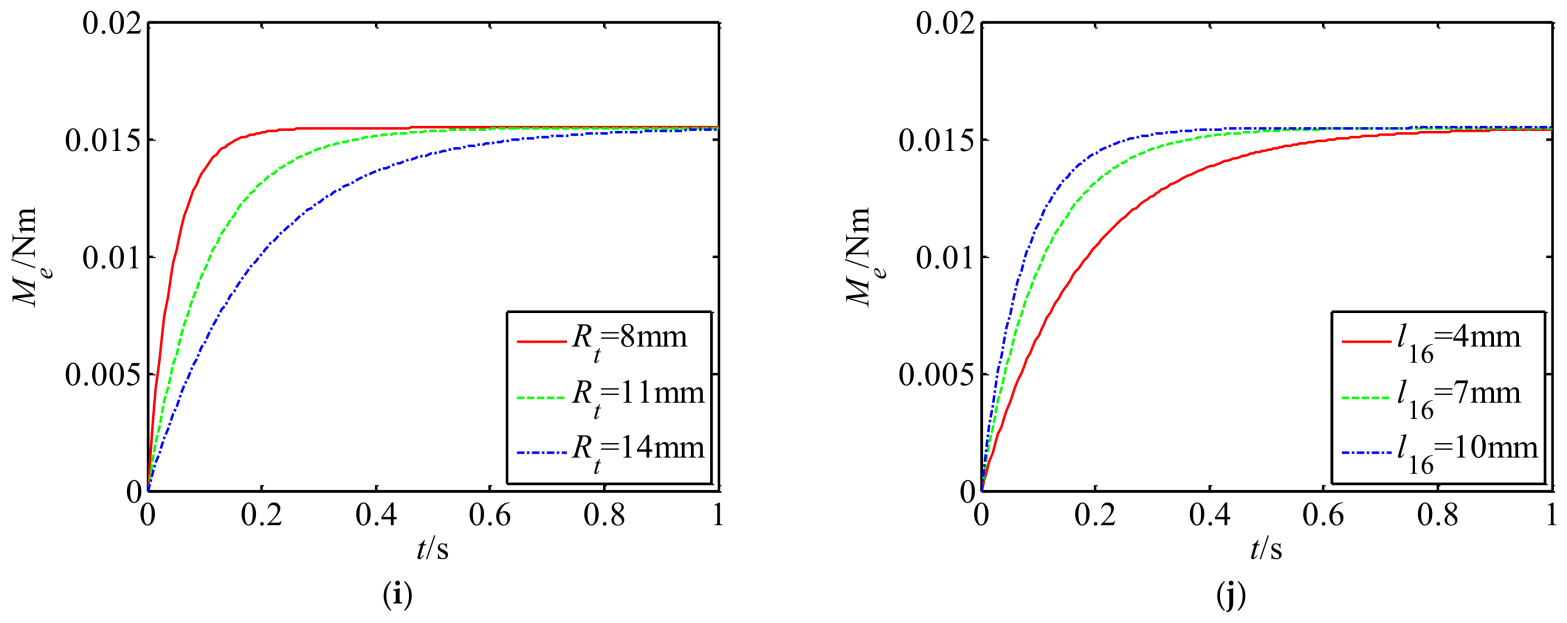
Step responses of output torque to input voltage under different parameters: (**a**) *N* changes; (**b**) *n_g_* changes; (**c**) *t_g_* changes; (**d**) *r*_2_ changes; (**e**) *α*_1_ changes; (**f**) *l*_12_ changes; (**g**) *R* changes; (**h**) *b*_12_ changes; (**i**) *R_t_* changes; (**j**) *l*_16_ changes.

The transfer function of the output torque to the input voltage of the non-contact piezoelectric motor can reflect the dynamic response of the system. To more clearly study the dynamic response of the system, the related parameters were substituted into the transfer function of the system to investigate its step responses (see Figure 11). Figure 11 reveals the following.

(1) The response time and response gain of the system are affected by the number of coil turns *N* and the resistance *R* of the winding. With the increase in the number of turns, the gain increases, but the response time becomes longer. Moreover, as the resistance of the winding increases, the response time decreases, but the gain decreases.

(2) The number *n_g_* of silicon steel sheets, the thickness *t_g_* of a single silicon steel sheet, the hollow rotor radius *r*_2_, the air-gap length *l*_12_, and the width *b*_12_ of the magnetic circuit greatly influence the response gain but have no effect on the response time. As the number of silicon steel sheets, the thickness of a single silicon steel sheet, the hollow rotor radius, and the width *b*_12_ of the magnetic circuit increase, the response gain increases. Moreover, as the length of the air gap increases, the gain decreases.

(3) The total coil length and average coil radius of the winding have a great influence on response time, but no effect on the response gain. The response time increases with the increase in the total coil length and the average radius of the winding.

Among these parameters, *N*, *n_g_*, *t_g_*, *r*_2_, *l*_12_, *b*_12_, *R*, *l*_16_, and *R_t_* have significant effects on the step responses of the system, while *b*_11_, *b*_14_, *b*_15_, *μ*_1_, *μ_r_*, *l*_13_, *l*_14_, *h*, and α_1_ have little effect on the step responses of the system.

In summation, changing the mechanical and electrical parameters of the system can improve the response performance of the system, but the response speed and steady-state amplitude cannot be significantly improved. Therefore, it is necessary to design a controller to improve the response characteristics of the system.

### 6.4. PI Control of the System

**Figure 12:**
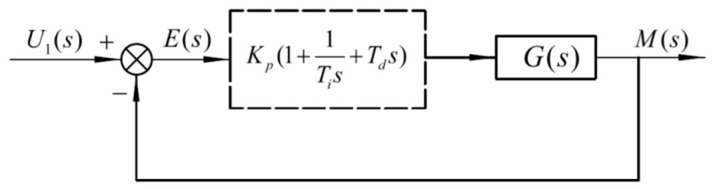
Schematic diagram of PID control.

Figure 12 exhibits the schematic diagram of the proportional-integral-derivative (PID) control system. PID control is a linear control method in which the difference between the actual output and input of the system is the object to be controlled. The PID controller converts the deviation signal generated by the system into the control quantity according to the linear adjustment of proportional, integral, and differential links. The relationship between the PID control input and output is

(34) $$m(t)=K_{p}e(t)+\frac{K_{p}}{T_{i}}\int_{0}^{t}e(t)dt+K_{p}T_{d}\frac{de(t)}{dt},$$

where *K_p_* is the gain of the proportional amplifier, *T_i_* is the gain of the integral amplifier, and *T_d_* is the gain of the differential amplifier.

**Figure 13:**
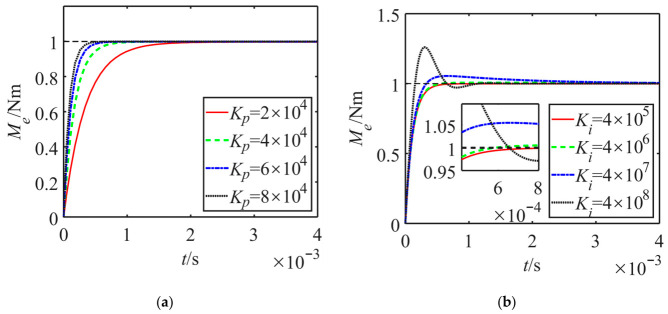
Step responses of the motor with proportional amplifier and integral amplifier: (**a**) with proportional amplifier; (**b**) with integral amplifier.

In the Simulink environment, the PI controller parameters are tuned using a heuristic trial-and-error method based on the system step response. Figure 13a exhibits the step response of the non-contact piezoelectric motor after the addition of a proportional amplifier.

Figure 13a reveals that the addition of the proportional link increases the response speed of the system. With the increase in the proportional gain coefficient, the response time of the system is shortened. When the proportional coefficient increases to greater than *K_p_* = 6 × 10^4^, the system performance cannot be significantly improved by increasing the proportional coefficient. Thus, the optimal proportional gain coefficient is considered to be *K_p_* = 6 × 10^4^.

On the basis of proportional control, the step response of the system after the addition of an integral amplifier is exhibited in Figure 13b.

Figure 13b indicates that the response speed of the non-contact piezoelectric motor is improved by adding the integral control link. As the integral gain coefficient increases, the response time of the system decreases, but the overshoot appears and increases. To avoid excessive overshoot, the optimal integral gain coefficient is considered to be *K_i_* = 4 × 10^6^.

After the addition of the differential amplifier, the change in the differential gain coefficient has little influence on the step response of the system. Therefore, the system does not require the addition of the differential link (the differential gain coefficient is considered to be *K_d_* = 0).

In summation, the controller required by the non-contact piezoelectric motor is a PI controller, the transfer function of which is

(35) $$G_{c}(s)=K_{p}+\frac{K_{i}}{s}.$$

The closed-loop transfer function is

(36) $$G_{PI}=\frac{G_{c}G}{1+G_{c}G}=\frac{K(K_{p}s+K_{i})}{Ts^{2}+(KK_{p}+1)s+KK_{i}}.$$

The closed-loop characteristic equation of the system is

(37) $$Ts^{2}+\left(KK_{p}+1\right)s+KK_{i}=0.$$

According to the Routh-Hurwitz criterion, since the system time constant *T* > 0, the system gain *K* > 0, and both the selected proportional gain and integral gain are greater than zero, all coefficients in Equation (37) are positive. Therefore, the chosen PI parameters satisfy the stability condition.

**Figure 14:**
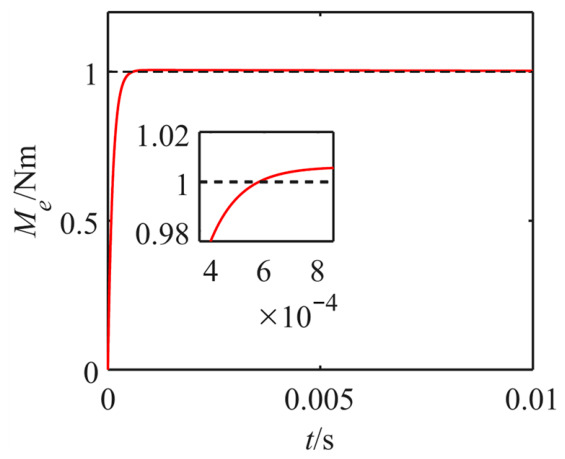
Step response of the closed-loop system with the PI controller.

Figure 14 exhibits the step responses of the closed-loop control system with the PI controller, and Table 1 reports the dynamic performance parameters of the system before and after the addition of the PI controller.

The response speed and steady-state amplitude of the step signals are greatly improved by the PI controller, and the overshoot of the system is controlled within an allowable range. Ultimately, a good dynamic response is achieved.

### 6.5. Output Torque Fluctuation and Compensation

**Figure 15:**
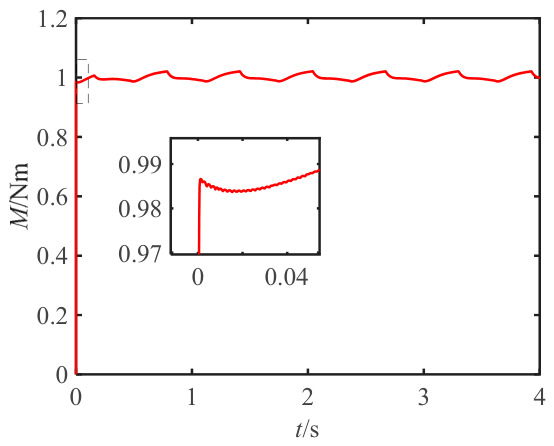
Step response of the electromagnetic torque.

By substituting the parameters reported in Table 2 and Table 3 into Equation (36), the step response of the electromagnetic torque of the non-contact piezoelectric motor can be determined, as presented in Figure 15.

Figure 15 reveals that the closed-loop control system of the non-contact piezoelectric motor system has a fast response speed, but periodic fluctuations occur. Thus, the design of the PI controller cannot eliminate the interference of the disturbance signal to the drive system.

**Figure 16:**
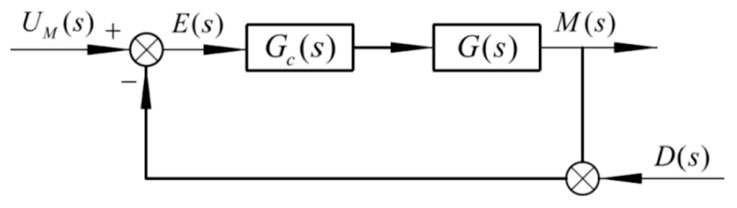
Control model of the voltage compensation.

Therefore, a compensating voltage is required to remove the torque fluctuation. Figure 16 presents a control model of voltage compensation for the disturbance. To obtain the compensation voltage signal that needs to be input, the inverse of the response caused by the torque fluctuation must be added to the input signals. The transfer function from the disturbance signal to the output voltage is

(38) $$G_{D}(s)=\frac{M_{d}(s)}{D(s)}=\frac{-G_{c}(s)G(s)}{1+G_{c}(s)G(s)}.$$

From Equation (38), the fluctuation voltage signals *M_d_*(*t*) can be obtained. Thus, the input signal of the system can be determined as

(39) $$U_{M}=U_{1}+M_{d}.$$

**Figure 17:**
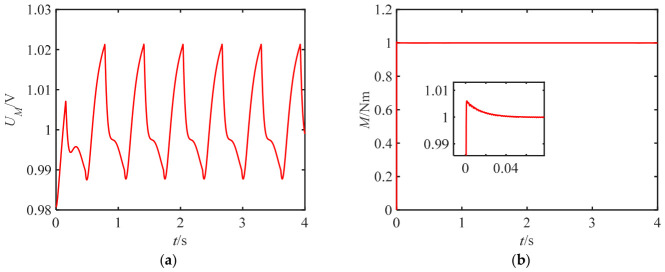
Compensation signal and torque response: (**a**) compensation signal; (**b**) torque response.

The compensation signal is given in Figure 17a, and the output torque of the system is given in Figure 17b.

The results indicate that the torque response of the motor system is rapid and the torque fluctuation is also removed. Thus, a good dynamic response is achieved.

## 7. Conclusions

In this paper, a novel non-contact piezoelectric motor modulated by electromagnetic force was proposed. The calculation model of the magnetic forces of the motor was deduced, and the calculated equations of the magnetic driving torque, the magnetic keeping torque, the total torque, and the torque fluctuation of the motor were presented. The transfer functions of the motor torque and fluctuation compensation control with a PI controller were also provided. Using these equations, the effects of the system parameters on the system gain and time constant were investigated. Moreover, the step responses of the motor torque were analyzed, as were the step responses of the closed-loop control system with a PI controller. Finally, the torque fluctuation of the motor and its compensation control were studied. The results of this research demonstrate the following.

(1) The number of coil turns, the radius of the rotor, the air gap length, the magnetic circuit width, the average magnetic circuit length, and the slip angle have an influence on the system gain. When the number of coil turns increases from 100 to 400, the system gain rises from 0.0039 to 0.062, representing an approximate 16-fold increase. When the magnetic circuit width *b*_12_ increases from 5 mm to 25 mm, the system gain rises from 0.0067 to 0.029, representing an approximate 4.3-fold increase. With the increase in the radius of the rotor, the system gain first increases, reaches a peak value at *r*_2_ = 32.42 mm, and then decreases. When the air gap length increases from 0.2 mm to 1 mm, the system gain decreases from 0.049 to 0.0032, representing a decrease by a factor of approximately 15.

(2) A smaller time constant can be obtained by decreasing the number of coil turns, the average radius of the winding, and the relative permeability of the silicon steel sheet; a smaller time constant can also be obtained by increasing the resistance of the winding coil, the total length of the coil, and the thickness of the winding coil.

(3) The response speed and steady-state amplitude of the step responses of the motor torque are greatly improved by the PI controller, and the overshoot of the system can be controlled within the allowable range.

(4) Simulation results demonstrate that, via the use of the PI controller and feedback compensation signals, the torque response of the motor system is rapid, and the torque fluctuation is eliminated. Ultimately, a good dynamic response is achieved.

## Figures and Tables

**Table 1:** Performance comparison before and after the addition of the PI controller.

-	Delay Time *t*_*d*_ (s)	Rise Time *t*_*r*_ (s)	Peak Time *t*_*p*_ (s)	Overshot *σ*_*p*_ (%)	Adjust Time *t*_*s*_ (s)
-	0.0723	0.2289	0.7826	0	0.4068
PI	7.9 × 10^−5^	2.447 × 10^−4^	8.2 × 10^−4^	0.7%	4.13 × 10^−4^

**Table 2:** Parameters of the keeping torque modulation mechanism.

*N*	*I* (A)	*μ*_0_ (H/m)	*α* (rad)	*U* (V)	*R* (Ω)	*S*_21_ (mm^2^)	*S*_22_ (mm^2^)	*S*_24_ (mm^2^)	*S*_25_ (mm^2^)	*r* (mm)
200	0.2	4π × 10^−7^	0.005	12	50	51	77.73	45.91	14	13.5
*μ*_1_	*μ*_2_	*μ_r_*	*h* (mm)	*ρ_R_* (Ω/m)	*R_t_* (mm)	*l*_26_ (mm)	*l*_21_ (mm)	*l*_22_ (mm)	*l*_23_ (mm)	*l*_24_ (mm)
4000	1	4000	3	1.7 × 10^−8^	6	4	24.21	0.5	1.5	4

**Table 3:** Parameters of the driving torque modulation mechanism.

*N*	*I* (A)	*μ*_0_ (H/m)	*α* (rad)	*U* (V)	*R* (Ω)	*n*_g_	*t*_g_ (mm)	*r*_2_ (mm)	*b*_11_ (mm)	*b*_12_ (mm)
200	0.2	4π × 10^−7^	0.021	12	50	30	0.35	17	2	12
*b*_14_ (mm)	*b*_15_ (mm)	*h* (mm)	*μ*_1_	*ρ_R_* (Ω/m)	*R_t_* (mm)	*l*_16_ (mm)	*μ_r_*	*l*_12_ (mm)	*l*_13_ (mm)	*l*_14_ (mm)
8	5	6.5	4000	1.7 × 10^−8^	11	7	7000	0.5	2.5	10

## Data Availability

The original contributions presented in this study are included in the article.

## Appendix A

The input voltage signal of the driving torque modulation mechanism *U*_1_(*t*) is from Equation (18)

(A1) $$U_{1}\left(t\right)=\frac{U}{2}+\frac{2U}{\pi }\sum_{n=1}^{\infty }\left[\frac{{\left(-1\right)}^{n-1}}{2n-1}\cos\left(2n-1\right)\omega t\right].$$

The electromagnetic modulation circuit consists of winding resistance and inductance, so it can be equivalent to a resistor–inductor (RL) circuit. According to the voltage relationship in the series circuit, it is known that

(A2) $$U_{1}\left(t\right)=L\frac{di_{e}}{dt}+Ri_{e},$$

where *i_e_* is the current in the driving torque modulation mechanism, *L* is the circuit inductance, and *R* is the circuit resistance.

Then, the general solution of Equation (A2) is

(A3) $$i_{e}\left(t\right)=e^{-\int \frac{R}{L}dt}\left(C+\int \frac{U_{1}\left(t\right)}{L}e^{\int \frac{R}{L}dt}dt\right)=e^{-\frac{R}{L}t}\left(C+\int \frac{U_{1}\left(t\right)}{L}e^{\frac{R}{L}t}dt\right).$$

The substitution of Equation (A1) into Equation (A3) yields the following

(A4) $$\begin{array}{c} i_{e}\left(t\right)=Ce^{-\frac{R}{L}t}+\frac{U}{2R}+\sum_{n=1}^{\infty }\frac{{\left(-1\right)}^{n-1}2U}{\left(2n-1\right)\pi \left(R^{2}+{\left(2n-1\right)}^{2}\omega^{2}L^{2}\right)}\left[R\cos\left(2n-1\right)\omega t+\right.\\ \left.\;\;\;\;\;\left(2n-1\right)\omega L\sin\left(2n-1\right)\omega t\right]. \end{array}$$

From the initial condition *i_e_* = 0 at *t* = 0, the following can be obtained

(A5) $$C=-\frac{U}{2R}-\sum_{n=1}^{\infty }\frac{{\left(-1\right)}^{n-1}2UR}{\left(2n-1\right)\pi \left(R^{2}+{\left(2n-1\right)}^{2}\omega^{2}L^{2}\right)}.$$

Thus, the dynamic current in the driving torque modulation mechanism is

(A6) $$\begin{array}{c} i_{e}\left(t\right)=\frac{U}{2R}\left(1-e^{-\frac{R}{L}t}\right)+\sum_{n=1}^{\infty }\frac{{\left(-1\right)}^{n-1}2U}{\left(2n-1\right)\pi \left(R^{2}+{\left(2n-1\right)}^{2}\omega^{2}L^{2}\right)}\left[R\cos\left(2n-1\right)\omega t+\right.\\ \;\;\;\;\;\left(2n-1\right)\omega L\sin\left(2n-1\right)\omega t-Re^{-\frac{R}{L}t}], \end{array}$$

which is Equation (21) in the main text.
